# The Intersections between Sociology and STS: A Big Data Approach

**DOI:** 10.1177/07311214231167170

**Published:** 2023-05-11

**Authors:** Maria Amuchastegui, Kean Birch, Wolfgang Kaltenbrunner

**Affiliations:** 1 York University, Toronto, ON, Canada; 2 Leiden University, Leiden, The Netherlands

**Keywords:** science, knowledge, and technology, science and technology studies (STS), history of sociology, Big Data, methodology

## Abstract

This paper charts the changing intersections between sociology and science and technology studies (STS) using computational textual analysis. We characterize this “quali-quantitative” approach as a Big Data method, as this calls attention to the commixture of textual and numeric data that characterizes Big Data. The term Big Data, too, calls attention to the increasing privatization of both data and data analytics tools. The data mining was done using a commercial analytics tool, IBM SPSS Modeler, that to the best of our knowledge has not yet been used for STS or sociological research. The identification of intersections occurred as part of a larger project to analyze political-economic and epistemic changes within STS, focusing on academic publishing. These epistemic changes were identified qualitatively, through 76 interviews with STS scholars, and quantitatively, through a computational analysis of three decades of STS journals (1990–2019).

## Introduction

Origin stories are necessarily partial and arbitrary, yet genealogies of science and technology studies (STS) often begin with sociology (Kreimer 2017; Law 2008; Pinch 2007; Roosth and Silbey 2009; Vinck 2016). These accounts sometimes situate the birth of STS in the 1960s and 1970s in the Sociology of Scientific Knowledge (SSK) (e.g., Rohracher 2015). Blurring the Popperian distinction between science and non-science, SSK scholars provided social explanations for both “erroneous” and “correct” science (Bloor 1976). Some genealogies start earlier, in the 1940s, with the functionalist sociology of Robert K. Merton (1973), who posited the existence of institutional norms that make science possible (e.g., Hess 2012). Either way, it is clear that STS has been strongly influenced by the diverse methods of sociology: for example, Merton and SSK often relied on historical sociological approaches; the Empirical Program of Relativism (EPOR) used qualitative interview methods; and actor-network theory drew upon ethnomethodology and ethnography (Collins 1981; Latour and Woolgar [1979] 1986; Magaudda 2014).

Despite the evident alignment between sociology and STS, differences persisted, particularly after the STS turn to ontology (Mol 1999). According to John Law (2008), for example, sociology ascribes causality to macro-social categories, conceiving of them as determinants of phenomena like inequality, while for some STS scholars—particularly those who subscribe to an empirical, material form of post-structuralism—the social is an effect of relationality (Latour 2000; Law 2008). That said, the recently emerging sociological study of Big Data represents a possible site for potential rapprochement, reflecting commonalities between digital sociology and the STS subdiscipline of scientometrics, known for the application of quantitative methods to scientific literature (Wyatt et al. 2017). With this emerging interest in Big Data, the application of quantitative methods to scientific literature has significantly expanded, offering new methods for exploring the production, constitution, and legitimation of the knowledge that underpins STS.

In this paper, we use computational textual analysis to outline and analyze the intersections between sociology and STS as they have evolved over time. The identification of these intersections occurred as part of a broader project exploring the political-economic and epistemic changes within journal publishing in the field of STS (see Kaltenbrunner et al. 2022). We analyze these political-economic and epistemic changes in two ways: qualitatively, through 76 interviews with STS scholars; and quantitatively, through a computational analysis of the evolution of theoretical topics, perspectives, and debates in three decades of STS journal publishing (1990–2019). We analyzed theoretical trends in the following “core” STS journals: *Social Studies of Science; Science, Technology and Human Values; Science as Culture; Social Epistemology; Science and Technology Studies; East Asian Science and Technology Studies; and Engaging Science and Technology Studies*. We identified theoretical trends by building a large data set of journal articles and “scraping” them for theoretical terms. This approach has been used to study other disciplines, including the philosophy of science, history, and classics (Malaterre, Chartier, and Pulizzotto 2019; Malaterre, Pulizzotto, and Lareau 2020; Martin 2019; Mimno 2012; Peirson et al. 2017). To the best of our knowledge, this approach has not been used for STS or sociology.

Through our analysis, we seek to answer the following questions: Which sociological concepts are evident in the seven core STS journals between 1990 and 2019? How did the frequency with which these concepts occur change over time? How have the intersections between sociology and STS evolved over time?

Our computational textual analysis was done using a commercial Big Data tool, IBM SPSS Modeler (Clark 2017). Although sociologists now use programming languages to identify concepts in large data sets, the tool that, historically, has most often been used for quantitative sociology is SPSS Statistics, the sister tool of SPSS Modeler. Emma Uprichard et al. (2008) relate that SPSS Statistics, originally known simply as SPSS, became the most popular statistical tool among sociologists soon after its invention in the late 1960s. They argue that SPSS Statistics is a Latourian inscription device that is “central to the experience of ‘becoming’ and ‘being’ a sociologist” (p. 606) and that it has changed the way quantitative sociology is done. SPSS Statistics data sets, created to train sociology students at the University of Surrey, were shared throughout the United Kingdom, where they served as Kuhnian examples of how to create normal sociological knowledge. In the 1980s, with the rise of the computer lab as a teaching space, quantitative sociology textbooks began to emphasize how to use SPSS Statistics, while de-emphasizing philosophical considerations of how quantitative knowledge is made. With the rise of Big Data, SPSS Statistics increasingly began to include statistical methods used in the commercial world, such as predictive analytics. Uprichard et al. argue that this commercial turn has epistemic and ontological implications for sociology.

Because SPSS Modeler often comes bundled with SPSS Statistics, which continues to be taught to many students in the natural and social sciences, it is already available to many sociologists. Moreover, it is designed to be used by those without knowledge of computer programming (Khabaza 1999). Nevertheless, despite the expressed interest of many sociology students in learning Big Data methods such as topic modeling (Mützel 2015), few social scientists seem to be aware of this tool. SPSS Modeler has been used in other academic disciplines, including psychology and medical research (e.g., Balogh 2010; Qiao et al. 2019). However, to the best of our knowledge, SPSS Modeler has not yet been used in STS or sociology. This paper will therefore double as an assessment of the usefulness of the SPSS Modeler tool for these disciplines.

The paper is organized as follows. We begin by outlining our theoretical approach. We include, in this section, debates in the STS and STS-adjacent literature on the use of quantitative methods, including Big Data methods, in the social sciences. Next, we describe the specific methods we used to extract text from journal articles and identify concepts in the text. Interspersed in this section, in a reflexive fashion, is a commentary on the “data journey” (Leonelli 2014) that we traversed. We include in this section an assessment of the usefulness of the SPSS Modeler text analytics tool for sociology and STS. We then discuss the findings of our quantitative analysis, comparing the quantitative findings with what the STS literature says about the changing relationship between sociology and STS. Where applicable, we also draw on qualitative materials collected during the project.

## Theoretical Approach

We characterize our approach as a Big Data method, as this appellation calls attention to the fact that Big Data commixes textual and numeric data (Swan 2013). Tommaso Venturini and Bruno Latour (2010) argue that the methods used to analyze Big Data must account for its dual nature, citing network diagrams as an example of what they call a “quali-quantitative” approach. David Moats and Erik Borra (2018) suggest modulation sequencers as another example of a quali-quantitative approach. Christopher A. Bail (2014) argues that, to track cultural changes in Big Data, the computational methods of computer science must be combined with a qualitative analysis that is informed by the expertise of social scientists. In our case, we use line charts, a quantitative tool, to visualize a textual data set obtained via a data mining algorithm. Data mining is quali-quantitative in that it entails the mining of textual data, which can then be summarized numerically. Line charts are among the oldest data visualizations, but have more recently become associated with Google ngrams (Bowker 2014; Rosenberg 2013).

We use the term Big Data, moreover, as it draws attention to the increasing privatization of both data and data analytics tools. Big Data connotes big industry and private interests; many scholars have noted that data, far from being open, have become increasingly enclosed (Muellerleile 2017). Rob Kitchin (2014) adds that data analytics tools, too, are becoming increasingly privatized. Unlike the similarly named SPSS Statistics, which originated in academia (Uprichard et al. 2008), SPSS Modeler originated in private industry (Khabaza 1999). SPSS Modeler, moreover, is made by IBM, a company which once dominated the tech sector so much that it was referred to as Big Blue (Campbell-Kelly et al. 2014). IBM itself characterizes its text analytics offering as a Big Data tool. In a sales manual for SPSS Modeler, recently posted on the IBM Web site, the term “big data” is used thirteen times (IBM Corporation 2021). Although we call attention to what makes Big Data methods different, we hasten to add that some of the claims about the novelty of Big Data are exaggerated. Noortje Marres (2017) historicizes digital sociology, pointing out that digital data, as well as the methods used by sociologists to study digital data, are not new. Sabina Leonelli (2014) relates that scientists have been working with large data sets for centuries. We concur, however, with the many scholars—Marres and Leonelli included—who acknowledge that something new is afoot.

As an object of study and quantitative method, Big Data differs from earlier forms of quantification in the social sciences. As Theodore M. Porter ([1995] 2020) argues, the quantitative methods taken up by postwar sociologists signaled an aspiration toward democracy and open knowledge. In contrast, Big Data often depends on data sets mostly owned by corporations, which also make the analytics tools with which these data sets can most readily be analyzed (Kitchin 2014). Similarly, whereas the use of traditional quantitative methods signaled a belief in the scientific method, the proponents of Big Data claim that it provides direct access to knowledge, and thus obviates the need for the scientific method (Anderson 2008). As Kitchin (2014) points out, this claim is convenient for the makers of commercial analytics tools. Big Data therefore certifies the objectivity of the knowledge that it encloses.

To derive knowledge from Big Data and to act on this knowledge, therefore, requires recourse to the very categories that, according to Bruno Latour (2005), are contingent and unstable, being constantly in a process of formation and reformation. Latour argues that Big Data methods constitute a novel statistical instrument and that they demonstrate his claim—drawing upon Gabriel Tarde—that the categories of “society” and “social” do not represent “a kind of material or domain” but rather a process of “assembling” (Latour 2005: 1; also Latour 2002, 2016; Latour et al. 2012). Geoffrey Bowker argues, *contra* Latour, that although these categories are imperfect, we live in a world in which such categories have material effects (Bowker 2014; Bowker and Star 1999). As Bowker points out, the insistence that correlation in Big Data is enough for social understanding, and that we thus do not need to discern causation, will diminish our understanding of the world.

In a prescient 2007 article, Mike Savage and Roger Burrows (2007) raised the alarm about Big Data and its methods, warning that the expertise of sociologists is at stake (Burrows and Savage 2014; Savage and Burrows 2007, 2009). Savage and Burrows argue that, as a result of what they term “transactional data,” sociology had lost jurisdiction over the social. Because we live in the age of “knowing capitalism,” corporations in the course of doing business gather and analyze transactional data about people. Corporations then analyze these data using novel methods such as cluster diagrams, a type of data visualization. As a result, it is corporations—and not sociologists—that now have privileged insight into the social world. Furthermore, Savage and Burrows (2007) claim, controversially, that the dominant methods of sociology—specifically, sample surveys and interviews—are not relevant outside sociology.

The “coming crisis,” as described by Savage and Burrows (2007), was a crisis of expertise. As Porter ([1995] 2020) relates, the postwar quantification of the social sciences was occasioned by a similar crisis. According to Porter, the turn to quantification was primarily a U.S. phenomenon, and it was caused by the climate of distrust that was pervasive in the political sphere. In the Depression era, American bureaucrats were able, like their European counterparts, to exercise personal judgment in deciding which public works projects to fund. In the postwar era, however, a climate of distrust prevailed. As a result, American bureaucrats began to use quantified decision criteria, such as cost-benefit analysis and risk analysis, as technologies of trust. This use of quantification as a technology of trust spread from U.S. government agencies to certain academic disciplines, including sociology. This distrust was allied, too, with neoliberal economists who opposed regulation. “It is no accident,” says Porter ([1995] 2020: 199), “that the move toward the almost universal quantification of social and applied disciplines was led by the United States, and succeeded most fully there.”

For Porter ([1995] 2020), quantification functions as a technology of trust because it is perceived to be objective. In other words, numbers connote rigor, neutrality, and fairness. Porter stresses that the status of quantification as a social technology does not imply that quantification has no basis in reality. Rather, it means that the invocation of numbers to signal objectivity is a social process. Objectivity, in turn, is historically situated (Daston and Galison 2021). For Enlightenment philosophers, the rational self was the source of objective knowledge. For Karl Pearson—who was a socialist and a positivist, and who was a founder of modern statistics—it is quantification that is the source of objective knowledge (Porter 2006). Pearson believed that reasoning in the aggregate implies a renunciation of the self, for the good of society.

Porter ([1995] 2020) refers to the mindless following of rules—whether by a human or by a machine—as mechanical objectivity. Porter implies that mechanical objectivity can be a good thing, as it tends toward democracy and open knowledge. Whereas a reliance upon the subjective judgment of experts can lack transparency, the quest to implement mechanical decision-making aspires to a transparency that subjective judgment lacks. The faceless civil servants who standardized the formula for cost-benefit analysis were conforming to the Weberian ideal of the dutiful bureaucrat. In the process, they created knowledge that is supposedly impersonal, objective, and open. Open knowledge, as Porter reminds us, is necessary to the formation of Jürgen Habermas’s public sphere.

If open knowledge is necessary to the creation of a public sphere, the perceived objectivity of knowledge is necessary to its epistemic legitimation. The epistemic and ontological claims about Big Data are often hyperbolic. For example, Chris Anderson (2008) famously claimed that Big Data speaks for itself, thus rendering the scientific method and theory obsolete. What makes Big Data qualitatively different, according to Anderson, is that it includes everything—the entire population—rather than a mere sample. Leonelli (2014) points out that Big Data data sets do not, in practice, contain all data. Moreover, decisions about which data points to include are based on political and economic decisions. Leonelli (2014) and Geoffrey C. Bowker (2014) concur that Big Data data sets do not materialize from the ether, but require work to produce. Leonelli adds that data never speaks for itself, but is necessarily interpreted through the lens of existing conceptual scaffolding. In other words, conceptual categories exist, and they matter.

For Bruno Latour (2016), Big Data reveals that the distinction between qualitative and quantitative sociology is false. According to Latour, the resurgence of interest in the nineteenth-century sociologist Gabriel Tarde is due not only to the “end of the social” but also to Tarde’s notion of the quantitative—a notion that anticipates the Big Data methods of today. In contrast with his contemporary Émile Durkheim, for whom the distinction between the individual and society is fundamental, and for whom humans are unique in nature, Tarde makes no such distinctions. Although Tarde famously said that “everything is a society” (Latour 2016:188), by this he meant that all aggregations are societies, whether they are composed of humans or molecules. Unlike the stable, human societies that underpin Durkheim’s social theory, Tardean aggregations are provisional and posthuman. What distinguishes the “societies” studied by natural scientists is that natural scientists grasp their objects of study from afar. As such, they focus on the larger structure rather than its individual components, and the (numeric) information that they obtain is necessarily partial. In contrast, because qualitative social scientists grasp human societies from the inside, the (textual) information that they obtain is much richer. Thus, the difference between quantitative and qualitative analysis is one of degree, not of kind. Tarde envisioned a day when scientific instruments would permit the collection of data that would validate his theory of the social. For Latour, the advent of Big Data means that this day has come.

We conclude our theoretical discussion by pointing out that our data set, like much Big Data, includes transactional data. The data set consists of text scraped from PDFs of entire journal issues, some of which were produced by learned societies. Although the bulk of the text consists of research articles, it also contains meeting proceedings and editorials. It is these meeting proceedings, in particular, that are transactional. The first learned journal, which was the house organ of the Royal Society, was similarly transactional. For this reason, it was aptly named the *Transactions of the Royal Society*. Marres (2017) notes that the transactional nature of digital data has been cited as evidence that it is “natural,” in contrast with the “artificial” data obtained via interviews or surveys. It is the supposed “natural” nature of Big Data that, according to its proponents, gives it epistemic legitimacy.

Our aim in the following is to examine the intersections of sociology and STS, drawing upon Big Data methods as an epistemic instrument. In drawing upon these methods, we address the following research questions:

**Research Question 1:** What sociological concepts underpin STS literature between 1990 and 2019?
**Research Question 2:** How did the frequency of these concepts change over time?
**Research Question 3:** How have the intersections between sociology and STS evolved?

## Methodological Discussion

### Background

To identify concepts in small data sets, sociologists generally use qualitative approaches, such as grounded theory (Walker and Myrick 2006). The problem with grounded theory, in particular, is that each researcher can end up eliciting a different set of concepts from the same data. The results are thus not replicable (Nelson 2020). Another problem with qualitative approaches is that they are time-consuming and thus not suitable for Big Data data sets. With the increased availability of large textual data sets, sociologists have begun to use quantitative approaches, such as computational algorithms, to identify concepts. Laura K. Nelson (2020) proposes computational grounded theory as a way to combine the strengths of grounded theory—its ability to discern meaning and to build theory—with the scalability of computational methods.

Although grounded theory is a qualitative method, it draws epistemic authority from quantitative methods. Unlike most qualitative methods, which are logico-deductive in that they attempt to force-fit the data to a pre-existing theory, grounded theory is inductive in that it seeks to allow concepts and theories to emerge “naturally” from the data (Walker and Myrick 2006). Grounded theory thus arose as a way to make qualitative sociology more “scientific” by making it more like quantitative sociology.

From an STS perspective, the seeming objectivity of computational results is an effect of the status of computational tools as “black-boxed” technological artifacts. A black box, in the parlance of computer science and engineering, is a device that receives input and emits output, but whose inner workings remain opaque. Madeleine Akrich (1992) argues that only stabilized, black-boxed technologies can serve as sources of objective knowledge. In contrast, non-stabilized technologies reveal the contingent choices made by their designers, and thus do not serve as sources of objective knowledge.

For Nelson (2020), it is the reproducibility of computational results that makes them more scientific, and therefore more objective, than results obtained through qualitative analysis. As Steven Shapin and Simon Schaffer ([1985] 2011) relate, the use of reproducible results to create an aura of scientificity was historically contingent. By writing up scientific experiments in such a way that their readers could later reproduce the experiments, early modern experimenters turned their readers into “modest witnesses” who could attest to the objectivity of the knowledge produced.

Although programming languages, like commercial software tools, can be used to create reproducible results, commercial tools tend to be easier to use. Programming languages have a steep learning curve, and thus are not accessible to the vast majority of sociologists who lack a background in computer science. The suitability of commercial tools for sociological research is therefore worth investigating. Because of the familiarity of many sociologists with SPSS Statistics, and because of the ability of SPSS Modeler to analyze large amounts of unstructured, textual data—a hallmark of Big Data—a methodological investigation of SPSS Modeler is particularly worthwhile.

In our use of the SPSS Modeler software tool, our approach differs from that advocated by Nelson (2020), who favors the sole use of programming languages. Our approach differs, too, in the means of cross-validation. Whereas Nelson advocates validating the results obtained by the first computational tool with a second computational tool, we checked our computational analysis of STS journal data against qualitative interviews conducted with contributors to the STS journals—interviews that had been conducted as part of the same research project. Our approach differs, finally, in its use of human expertise to categorize concepts. The SPSS Modeler tool does have the ability to automatically categorize concepts, but only in knowledge domains that matter in the commercial world, such as social media, marketing, customer service, or—the most recent addition to the tool—genomics. This supports the claim by Uprichard et al. (2008) that, despite the origins of SPSS in academe, the current makers of SPSS are more attuned to commercial interests than to the needs of social scientists.

As of the time of writing, users who are affiliated with a university can obtain a free license for SPSS Modeler through the IBM Academic Initiative. For users who are outside academia, however, it is an expensive tool. Training courses on how to use the tool are also expensive. Moreover, users with academic licenses do not have access to tech support, which can hamper their ability to use the tool. It is worth noting that IBM became synonymous with mainframe computers, despite not having pioneered the technology, because it had a marketing strategy of providing inexpensive mainframe computers to universities (Campbell-Kelly et al. 2014). It thereby created a generation of graduates who became familiar with IBM products and later recommended them to their employers. IBM seems to be adopting a similar strategy in bundling the SPSS Modeler tool, which is not well known among academics, with the SPSS Statistics tool, which is widely used in academic research.

### Approach

We employed a mixed-methods approach, using quantitative methods to identify concepts in a large data set, and then validating our results qualitatively, with a smaller, related data set. The first data set consisted of the full text of all journal articles from seven STS journals for the time period 1990–2019. We used a programming language, Python, to create this data set, and we used a commercial software tool, SPSS Modeler, to identify concepts within it. The second data set consisted of the transcripts of 76 interviews conducted with individuals who had contributed to the journals in some way. The interview subjects were identified through stratified sampling, according to which the population was divided into categories, with samples taken from each category (Blaikie and Priest [2009] 2019). The categories were gender; career stage (early career, mid-career, or late career); region (Europe, North America, Latin America, or Asia); and role (author, editor, reviewer, board member or publisher).

To create the first data set, we used the Python programming language to extract (“scrape”) the text of journal articles from the associated PDFs. Some of the PDFs could not be scraped, due to data quality issues. This happened much more often with the PDFs produced by the smaller, less well-funded journals, than with the PDFs produced by the major academic publishers. As Leonelli (2014) has shown, institutions with less funding are more likely to produce data sets with data quality issues, which means that these data sets are more likely to be excluded from the Big Data data sets that are used in open science.

We also used the Python programming language to add metadata—data about the data—to the data set, including the name of the journal in which the article appeared, the year of publication, and the number of characters in the article. As Leonelli (2014) has shown, the addition of metadata is a crucial step in the “data journey” that data sets must traverse in order to become Big Data. In the case of the biological sciences, data sets without metadata are much less likely to be used by researchers in other institutions. However, the addition of metadata requires time, resources, and specialized skills, which are not evenly distributed among institutions. This implies that data sets that originate in institutions with fewer resources are less likely to form part of the Big Data data sets that are used in open science.

To add metadata to a database record is to classify it. As Geoffrey C. Bowker and Susan Leigh Star (1999) have shown, classification implies value judgments and has material consequences. For example, the number of characters was included in the metadata, as this permitted the authors to determine, in most instances, if the PDF was a research article. Since we were interested in epistemic changes over time, we judged that this would be captured best in research articles rather than in editorials or book reviews. As such, our division of PDFs into research articles and non-research articles constitutes part of our analytical assumptions about the evolution of conceptual developments in STS.

We then used SPSS Modeler to “cleanse” the data and to inductively identify “concepts.” This resulted in over 100,000 concepts, including common nouns (such as “periphery”), proper nouns (such as “Latour”), and noun phrases (such as “actor-network theory”). We excluded concepts that appeared too infrequently (in fewer than 1 percent of the documents). This resulted in an initial list of about 17,000 concepts. We then manually reviewed the list and excluded words that were not meaningful, resulting in a final list of 1,794 concepts.

Finally, we used Excel to create exploratory data visualizations, enabling us to identify those concepts that had the most interesting trajectories. We selected dozens of candidate concepts from among the 1,794 that we had flagged for further analysis, and we visualized their trajectory with line charts. The charts showed, for each decade, the percentage of articles in which each concept appeared. Since the total number of articles published each decade has increased over time, we decided to show percentage values, rather than absolute values. The line charts enabled us to further winnow the list of selected concepts, by identifying those concepts that had the most interesting trajectory. We defined “interesting” in narrative terms, as an upward or downward trajectory that suggests a potentially compelling story.

## Findings: Analyzing the Intersections between Sociology and STS

In tracing the changing intersections between sociology and STS, we found that STS has become both more and less sociological over time. On the one hand, STS has become more sociological, in that sociology and anthropology have become the dominant disciplines within STS, which was originally conceived of as a confluence of the history, philosophy, and sociology of science (Fuller 2007). On the other hand, STS has become less sociological, in that the structuralist sociology that informed much early STS scholarship has given way, among many STS scholars, to a post-structuralist problematization of the social (Latour 2002; Law 2008).

To further complicate matters, determining the extent of the indebtedness of STS to sociology depends on the national context. John Law, writing about the relationship between STS and British sociology, argues that, for sociologists, macro categories such as race and class are foundational (Law 2008). In contrast, for STS scholars of a poststructuralist bent (Latour 2000), the social is an effect or assemblage of interactions. For Mario Biagioli (2009), sociology continues to be dominated by an Anglo-American philosophical emphasis on the human-nature divide, whereas STS increasingly draws inspiration from the posthumanism of continental philosophy. This ontological turn within STS thus signaled a shift away from sociology and toward other fields, especially anthropology. It is possible that this is the result of the institutional pressures on STS scholars, especially in the North American context, where STS became embedded more in anthropology departments rather than sociology departments. This came through in our qualitative interviews, with one informant noting,

If you look at the US, there are very few STS scholars in sociology departments. The other natural home is anthropology. And if you—and I think at the same time, when sociology was saying no, anthropology was saying yes. So if I think about—I can say if I look at some of the major STS scholars or anthropology departments, there are people in those anthropology departments who are STS scholars who come to 4S [Society for Social Studies of Science Conference]. So the relationship between STS and anthropology, anthropology was much more open, they took in these STS’ers, even if their PhD’s were in STS. (#68, Established Scholar, North America)

Another informant made a similar point:

And one of the issues that I’ve seen is that even the best students who have a PhD in STS have a lot of difficulty migrating into a disciplinary department. I think this pattern is especially strong in the more prestigious social science disciplines such as political science or sociology, with anthropology being a little more open. (#53 Established Scholar, North America)

In mapping these changing intersections between sociology and STS, our first order of business, then, was to determine what constitutes an intersection. We considered tracking mentions of canonical sociologists, such as Robert Merton, in the STS journals that were our data source. This smacked of citation analysis, which was not our focus. We considered tracking mentions of the discipline of sociology or the profession of sociologist. This seemed more promising, although such mentions seemed likely to be a measure of the institutional power of the discipline of sociology, rather than an expression of specific intellectual trends. We considered tracking mentions of data gathering methods that originated within sociology—specifically, interviews and surveys (Savage and Burrows 2007, 2009)—as opposed to data gathering methods that originated in other disciplines. This, too, seemed promising, although it hardly seemed to get at the heart of the matter. Finally, we considered tracking intellectual currents within STS that are indebted to sociology, the most well-known example being the sociology of scientific knowledge (SSK). This seemed imperative, although it begged the question of which intellectual currents within STS are indebted to sociology.

### Sociology as Discipline

**Figure 1.**
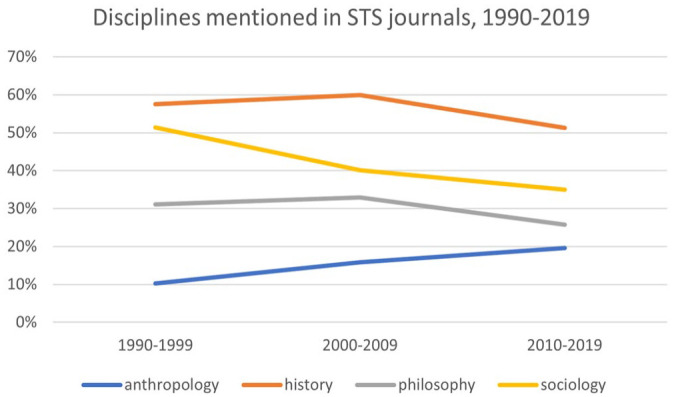
Disciplines mentioned in STS journals, 1990–2019. *Note.* STS = science and technology studies.

To determine the relationship between STS and sociology, writ large, we initially searched our data set for the disciplinary names of sociology, anthropology, history, and philosophy. Our findings showed that, in the pages of the core STS journals, the word “history” is consistently mentioned more often than the words “sociology,” “philosophy,” and “anthropology” across the three time periods (see Figure 1). Over time, moreover, there was a clear declining trend in references to history and philosophy, suggesting that STS has become more social scientific over time. What makes this more significant, for sociology, is that the terms history and philosophy are often used to refer to things other than academic disciplines, which likely accounts for a portion of the frequency of the occurrences of those terms.

**Figure 2.**
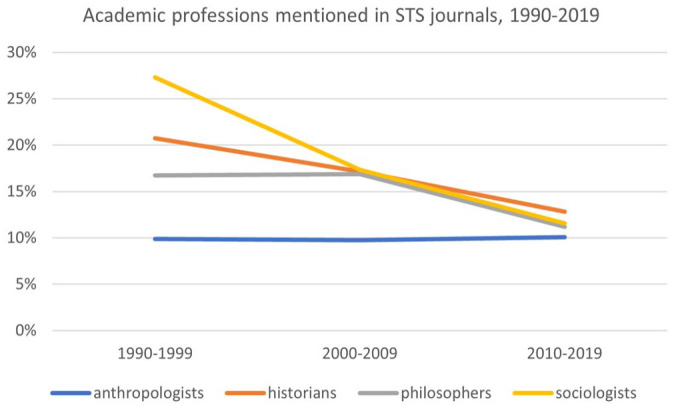
Academic professions mentioned in STS journals, 1990–2019. *Note.* STS = science and technology studies.

We then searched our data set for mentions of the occupational categories of sociologist, anthropologist, philosopher, and historian. In each instance, we searched for plural form of the term, as that was the form that had been identified by the concept identification algorithm as occurring more often than would be expected. This showed that, in STS journal articles, mentions of the plural noun “sociologists” have declined significantly, from a high of 27.29 percent in the 1990s to a low of 11.55 percent in the 2010s (see Figure 2). Mentions of philosophers and historians have also declined, but not by as much. Only anthropologists have held steady.

At first glance, these data seem to validate the claim that, in the field of STS, sociology has declined in importance (Biagioli 2009; Law 2008; Ruppert, Law, and Savage 2013). Conversely, a case could be made for the reverse. In the 1990s—the earliest decade captured in our data set—the sociological study of science was novel, and sociologists of science had less institutional power than historians and philosophers of science. The frequency with which sociologists are named in the pages of STS journals in the 1990s may, paradoxically, be a reflection of their status, among scholars of the sciences, as upstarts. By naming themselves, by calling out their status as outsiders, they distinguish themselves from the fields that were then hegemonic (Bourdieu 1984). According to this line of reasoning, the relative paucity of references to the profession of sociologist in the 2010s may be a measure of the institutionalization of sociology within STS. Having achieved institutional hegemony, sociologists no longer need to name themselves.

Interviews conducted with STS scholars lend support to both lines of reasoning. On the one hand, several scholars opined that STS has become less sociological, in the traditional sense. For example, one STS scholar, based in Europe and trained as a sociologist, decried this trend: “I’m a passionate sociologist,” they said,

and I don’t want to lose this sociological influence from STS . . . the kind of traditional sociological approach, yeah, I think it has become a little bit less sociological. I think it’s become a little bit more STSish. (#73 Mid-career Scholar, Europe)

In contrast, another STS scholar, trained as a historian and based in North America, lamented that historians have lost influence within STS and that sociologists have taken over: “History used to be more central when STS was founded in the mid to late 70s,” they said.

There has been some kind of, a bit of an alienation. I went to a [4S] meeting in New Orleans last year, and there were very few historians. . . maybe one can say that the field is [now] dominated by sociologists. (#39 Established Scholar, North America)

Despite this apparent decline, certain sub-disciplines within sociology have emerged as areas of interest with STS (see Table 1). In particular, the sociology of health is now mentioned in over 5 percent of STS journal articles. In addition, cultural sociology, economic sociology, political sociology, and the sociology of expectations are now mentioned in about 2 percent of articles. Finally, critical sociology and the sociology of work are now mentioned is about 1 percent of articles.

**Table 1.** Emerging Sociologies in STS.

Concept	1990–1999 %	2000–2009 %	2010–2019 %
Critical sociology	0.00	0.00	0.93
Cultural sociology	0.00	0.00	1.97
Economic sociology	0.00	0.00	1.56
Historical sociology	7.22	4.52	2.73
Medical sociology	2.29	2.20	2.63
New political sociology	0.00	0.00	1.49
New sociology	7.34	4.01	3.46
Political sociology	0.00	0.00	1.21
Sociological analysis	12.50	6.27	3.15
Sociological approach	2.18	1.36	0.00
Sociological explanation	2.98	0.00	0.00
Sociological perspective	2.98	0.00	1.62
Sociological perspectives	0.00	2.39	0.00
Sociological reconstructions	1.61	0.00	0.00
Sociological research	1.72	1.36	1.07
Sociological studies	5.39	1.62	0.00
Sociological theory	5.50	3.36	3.42
Sociologists	27.29	17.32	11.55
Sociology	51.38	40.14	34.95
Sociology of education	0.00	1.49	0.00
Sociology of expectations	0.00	1.29	2.28
Sociology of health	2.41	6.72	5.36
Sociology of knowledge	10.44	6.46	4.56
Sociology of mathematics	1.61	0.00	0.00
Sociology of monsters	2.98	3.36	2.70
Sociology of science	31.88	23.46	12.41
Sociology of technology	8.94	4.65	2.56
Sociology of translation	4.13	3.49	3.18
Sociology of work	0.00	1.68	1.11

### Sociology as Method

**Figure 3.**
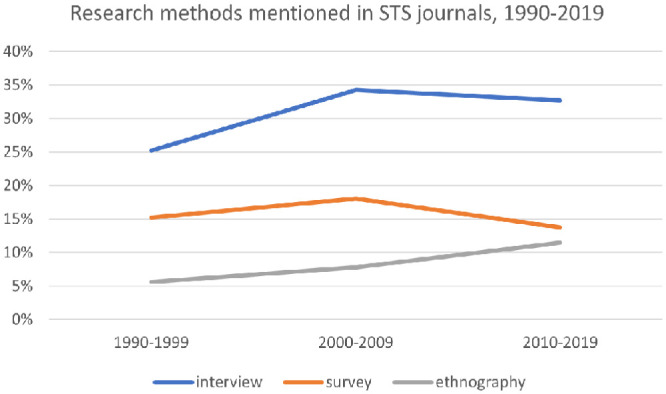
Research methods mentioned in STS journals, 1990–2019. *Note.* STS = science and technology studies.

Our data show that, methodologically, STS continues to be indebted to sociology. In particular, STS scholars primarily use interviews—a research method highly prevalent in sociology (Savage 2013)— to gather qualitative data. In the 1990s, a quarter (25 percent) of research articles in STS journals mentioned the word interview (see Figure 3). In subsequent decades, this increased to around a third—34 percent in the 2000s and 32 percent in the 2010s. Of course, the frequency of occurrences of the word interview in STS research articles does not map exactly to the frequency with which interviews were used by the authors to gather data. However, it represents a reasonable proxy for the importance accorded to the method.

Ethnography, another method used by STS scholars to gather qualitative data, also increased throughout the time periods captured in the data set. In the 1990s, the word ethnography was mentioned in 5.6 percent of STS research articles; by the 2000s, this had increased to 7.8 percent; and in the 2010s, it had increased again to 11.5 percent (see Figure 3). Although ethnography is most closely associated with anthropology (Wyatt et al. 2017), it is also used by many sociologists (Blaikie and Priest [2009] 2019). The most well-known STS scholar, Bruno Latour, famously used ethnography to observe scientists at the Salk Institute, observing them in the same manner that an anthropologist would study a group of people (Latour and Woolgar [1979] 1986). Latour himself identifies as a sociologist (Latour 2016).

Surveys, another research method closely associated with sociology (Savage and Burrows 2009), declined somewhat in importance over the time periods we considered. In the 1990s, the word survey was mentioned in 15.3 percent of STS research articles; in the 2000s, it peaked at 18 percent; and in the 2010s, it declined to 13.7 percent (see Figure 3). Depending on how they are designed, surveys can be a method for gathering quantitative data. Two of the STS scholars interviewed for this project pointed out that, whereas quantitative research continues to be important in sociology, few STS scholars do quantitative research. They speculated that this might be among the reasons that STS scholarship may not be well-received by traditional sociologists (#10 Established Scholar, Europe; #33 Established Scholar, Europe).

### Sociological Categories

Biagioli (2009) claims that STS has moved away from the structural sociology of Emile Durkheim, according to which the divide between society and the individual is key. For Law (2008), STS has become less sociological in the sense that macro categories—such as race, class or the social—matter less to STS scholars, particularly those of a postmodern bent. Steve Fuller (2007) claims that STS has lost its way, and it no longer has a sense of a public good, a *res publica*, for whom knowledge is created. Does the data support these claims?

**Figure 4.**
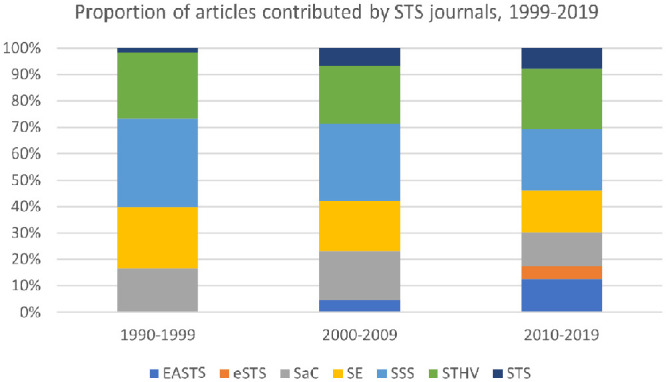
Proportion of articles contributed by STS journals, 1999–2019. *Note.* EASTS = East Asian Science, Technology and Society; eSTS = Engaging Science, Technology, and Society; SaC = Science as Culture; SE = Social Epistemology; SSS = Social Studies of Science; STHV = Science, Technology, & Human Values; STS = Science & Technology Studies.

Addressing these issues entails searching the data set for macro categories, such as society, culture, or the social. The first problem that arises is that several of the journals from which the data set was created have macro categories in their names. Specifically, *Social Studies of Science* and *Social Epistemology* have the word social in their title; *Science as Culture* has the word culture in its title; and *Science, Technology and Human Values* has the word human in its title. Journal titles appear in every PDF document from which the text was mined. As a result, the data set overstates the extent to which these macro categories appear in research articles. Moreover, due to the appearance of new journals, the proportion of articles contributed by those four journals declined somewhat, which accounts for some of the decline in mentions of macro categories (see Figure 4). However, since the journal names have remained consistent throughout the time period under study, and since the decline was not significant, it is safe to treat occurrences of macro categories in journal names as a constant that can be factored out. In other words, for macro categories that appear in journal names, the percentage value is overstated, but the trend is roughly correct. It is worth noting that the macro category that mattered most to Durkheim—society—does not appear in any of the journal titles. As such, the high percentage values for society are not overstated.

Another problem is that the macro category of “the social,” which is often invoked in the literature on sociology and Big Data, contains what is known as a stop word. Stop words are words, such as definite and indefinite articles (“the,” “a,” and “an”), that are ignored by natural language processing algorithms, as they are usually not meaningful. It would have been possible to configure the SPSS Modeler concept identification tool to make an exception in the case of “the social.” However, the tool would not have been able to distinguish between instances where “the social” is being used as a noun and instances where it is being used as an adjective, which would have defeated the purpose of making an exception. It is undoubtedly possible to create an algorithm that makes this distinction, but creating new algorithms was beyond the scope of our project. As a result, we decided to treat all occurrences of the word social as a permutation of the concept of the social.

**Figure 5.**
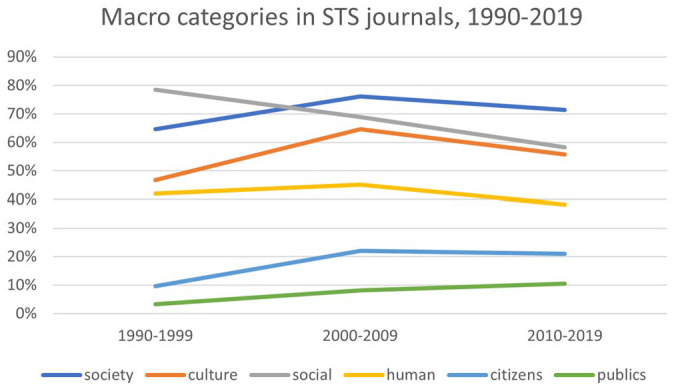
Macro categories in STS journals, 1990–2019. *Note.* STS = science and technology studies.

**Figure 6.**
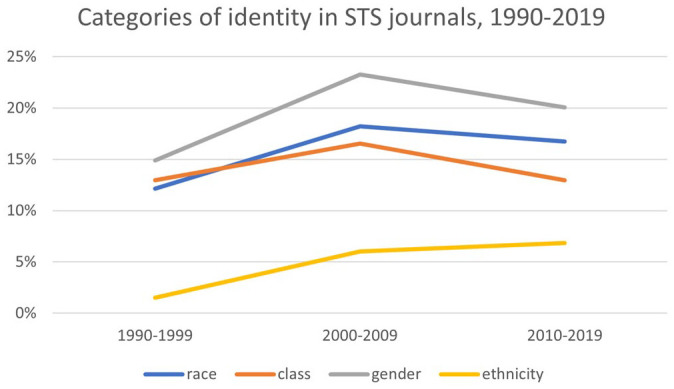
Categories of identity in STS journals, 1990–2019. *Note.* STS = science and technology studies.

Our data show three trends. First, most of the macro categories trended up from the 1990s to the 2000s, and then trended down from the 2000s to the 2010s. Included in this trend are most of the categories that relate to humanity as a whole: for example, society, culture, and human (see Figure 5). Also included in this trend are most of the macro categories that relate to individual identity: race, gender, and class (see Figure 6). This supports the claim that macro categories are increasingly less important to STS, especially categories like social, society, and culture, which intersect with sociology. It also shows that it was not always so. From the 1990s to the 2000s, macro categories actually increased in frequency. Indeed, the inclusion of sociological macro categories in many STS journal names shows that sociology is baked into the conceptual underpinnings of STS. The high percentage values for society—over 70 percent of STS journals articles in the 2010s contained the word society—indicate that macro categories still matter.

Second, macro categories that relate to the collective political and social action—citizens and publics—increased throughout the period under study, although they remained small in proportion to the other macro categories (see Figure 5). The fact that categories relating to political and social action occur proportionately less frequently than the other macro categories provides some support for Steve Fuller’s (2007) claim that STS, as a field, does not focus theoretically on social and political change. However, the consistent increase in these categories shows that this has been changing over time. The third and most striking trend is that the proportion of articles that contain the word *social* declined precipitously (see Figure 5). This, more than anything else, supports the claim that, within the pages of STS journals, the social has come to matter less over time.

### Sociology as Legitimation

The emergence of Big Data in STS journals occurred against the backdrop of a declining interest in the physical sciences like physics and chemistry, and an increasing interest in biology and biomedicine. For Geoffrey C. Bowker (2013:170), the current STS interest in hard data recalls its earlier preoccupation with the physical sciences:

In the old days of science studies, we used to worry about the supremacy of the hard sciences, which we rightly tied to the absolutism of the Christian theology they supplanted. Now we risk being in the grip of hard data.

The early STS literature emphasized the study of the physical sciences, such as physics, as these were seen as the “hard cases” that would conclusively prove that scientific knowledge is socially constructed (Bloor 1973; MacKenzie 1978). It was not until later that disciplines like biology and medicine came to be ascendant. In the 2010s, algorithms and Big Data began to be mentioned in STS research articles, heralding a return to an earlier preoccupation.

**Figure 7.**
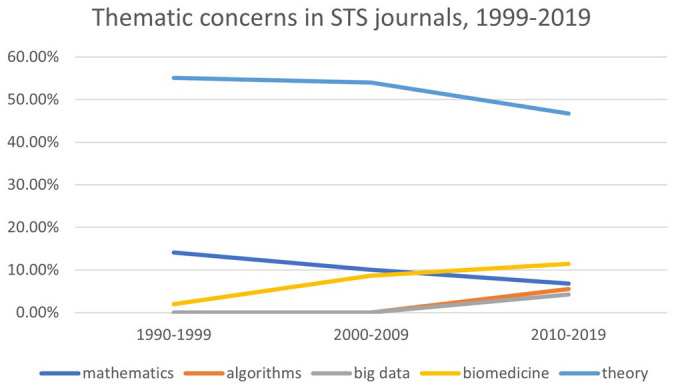
Thematic concerns in STS journals, 1999–2019. *Note.* STS = science and technology studies.

This trajectory can be discerned in the chart below (see Figure 7). In the 1990s, the word mathematics appeared in nearly 15 percent of STS journals. This does not imply, of course, that those articles were about mathematics; it merely indicates that numbers were evoked, like a talisman of objectivity (Porter [1995] 2020). The decline of the word mathematics is mirrored, almost exactly, by the rise of the word biomedicine. The terms big data and algorithm surge suddenly, *ex nihilo*, in the 2000s. For some informants, the emergence of new topics indicates a tendency to analyze new themes through the lens of existing STS theory. Indeed, as can been seen in Figure 7, the word theory was mentioned less often as time went by. In the words of one informant, “A lot of, you know, more thematic things like data, genomics and, yeah, those are clearly themes that are very prominent” (#61 Early Career Scholar, Europe).

Another possible interpretation of the increased interest in Big Data is that it provides the epistemic legitimacy of numbers. Porter (2020) would say that this recourse to quantification—as an object of study, if not as a method—is a sign of a weak discipline. Given the status of STS in the 1990s as an emergent discipline, and given the current STS ontological turn, this explanation makes sense. What the chart below does not show—for every data visualization conceals more than it depicts—is that the early STS study of the “hard sciences” was done through the lens of sociology. In particular, early STS scholars studied the hard sciences using not only the historical methods of sociologists such as Merton, but also the methods of empirical sociology. Just as quantification legitimized the beleaguered discipline of sociology in postwar America, so too did empirical sociology legitimize the nascent discipline of STS. What remains to be seen, however, is the role that the study of Big Data will play in the disciplines of STS and sociology. An informant pointed out that this might simply reflect funding priorities, rather than any significant epistemic shift in the field:

So, for example, in the year in which, you know, nanoscience was a thing you got a lot of papers on nanoscience. In the era in which the UK especially had buckets of money invested in human genome stuff, personalized genomics, that kinds of thing, we saw, again, tons of papers in that particular area. So you do see empirical trends that are—I think are mainly related to the funding trends. (#6 Mid-career Scholar, North America)

## Conclusion

In this paper, we have charted the changing relationship between STS and sociology from 1990 to 2019. We examined traces of this relationship in a large data set, a data set that was collated from the full text of all research articles that appeared in seven “core” STS journals during the time period under study. We confirmed our findings by analyzing a related data set, a data set consisting of interviews with STS scholars who had contributed to the journals in some way. We used a mixed-methods approach, combining qualitative analysis of the interviews with a computational textual analysis of the research articles. We performed the computational analysis with a commercial Big Data tool, SPSS Modeler. To our knowledge, this tool has not previously been used for sociological or STS research. We note that SPSS Modeler is the sister tool of SPSS Statistics, a tool that is widely used by quantitative sociologists and that, according to Uprichard et al. (2008), has changed the way quantitative sociology is done.

We found the SPSS Modeler tool to be useful but imperfect for our purposes, as the tool is more attuned to the needs of commercial customers than to the needs of social scientists. In particular, the tool was able to inductively identify STS and sociological concepts, but it was unable to categorize those concepts in a meaningful way. If technological artifacts perform social relations, then the inability of the SPSS Modeler tool to categorize social science concepts could be seen as an embodiment of the increasing privatization of knowledge-making practices. Uprichard et al. (2008) note that SPSS Statistics is increasingly oriented toward predictive analytics, a statistical method that is little used in most academic disciplines, but that is central to the new field of surveillance studies. As IBM notes in a blog post about the Manchester police, the SPSS Modeler tool can also be used for surveillance (Clark 2017). The ability of SPSS Modeler to be used for surveillance is an enactment of the relationship between private corporations and the surveillance state. The SPSS Modeler tool, designed to give private corporations and the carceral state privileged access to the social, is the technological incarnation of the “coming crisis” in sociology of which Savage and Burrows (2007) had warned.

To reveal the social relations inherent in a technological artifact is to pry open the black box. Dominique Vinck (2016) concurs with Sophia Roosth and Susan Silbey (2009) that it is the concept of the black box, applied to technological artifacts or scientific facts, that constitutes STS’s main contribution to sociology. But whereas sociologists tend to agree that technology is socially constituted, the extension of this analysis to scientific facts is more contentious. Indeed, in accounts of the relations between STS and adjacent disciplines, the Science Wars loom large (see Daston 2009; Jasanoff 2000). Since quantitative sociologists, in particular, view their work as scientific, they may be resistant to STS. As Latour (2000) quips, “Social scientists may be right, after all, in wanting to have no dealings with a field that destroys the scientificity of all the sciences by explaining all of them socially” (p. 111). The problematization of scientific facts is therefore a contribution by STS to sociology, as well as a reason for sociology to resist STS. As Pablo Kreimer (2017) notes, sociologists tend to be constructivists with respect to their own discipline, but positivists with respect to the natural sciences.

Although it was Latour who extended the metaphor of the black box from technological artifacts to scientific facts (Epstein 1996), Bruno Latour (2000) himself argues that the main contribution of STS to sociology is the extension of agency to nonhumans. From a Latourian perspective, our inability to coax the SPSS Modeler tool into producing the desired results reveals the agency of the tool. Latour (2000), who describes STS as “a minuscule subfield of sociology” (p. 107), says in the same breath that “STS is rarely read amongst mainstream sociologists” (p. 112) and that STS has nevertheless had a “shocking influence . . . on the natural and social sciences” (p. 120). This apparent paradox can be explained by what Kreimer (2017) calls the Latour effect. Latour’s influence on the social sciences is so outsized that he is often taken to stand for all of STS. Sociologists who disagree with Latour’s poststructuralist views may therefore be inclined to disregard the rest of STS.

Perhaps, though, it is difficult to clearly identify the intersections between sociology and STS because they are so bound up with one another. Leydesdorff (2022) describes STS as a liminal field that “developed in the overlaps at the margins of sociology with other disciplines” (p. 5). Roosth and Silbey (2009) point out that the precise contribution of STS to sociology is difficult to discern, as both fields have been influenced by, and continue to influence, similar intellectual currents. For Peter Dear and Sheila Jasanoff (2010), discussions of the relationship between STS and adjacent disciplines are misguided, as all disciplines are interdisciplinary. The account that emerges of the relationship between sociology and STS—as heterogeneous, unstable, and co-constitutive—is one that is consonant with STS theory. It also describes a productive way forward.
